# Popliteal Aneurism

**Published:** 1849

**Authors:** Daniel Brainard

**Affiliations:** Professor of Surgery in Rush Medical College


### ARTICLE VIII.

*Popliteal Aneurism. Rupture of the Sac. Ligature of the Femoral Artery. Recovery.* By Daniel Brainard, M.D. Professor of Surgery in Rush Medical College.

Catharine Driscoll, æt. 24 years, was seized about February 1st, 1849, during the night, with a severe hemorrhage, which was near proving fatal. She was seen by Dr. Chas Duck, who used a temporary dressing, and she was then carried to the Chicago private hospital, and came under my care.

The patient was of very limited intellect, and apparently of irregular life, and could give no other account of the disease except that she had been kicked by a cow behind the right knee when she was very young. On examination, the right femur was found to be affected with an ancient necrosis at its lower part, it being enlarged and irregular. There were numerous depressed cavities and three fistulous openings on the surface of the member. In the popliteal space was a fluctuating elastic tumor, about 2½ inches in diameter, pulsating, and giving the rasp sound. These latter signs were suspended when the femoral artery was compressed. The knee was anchylosed in the straight position, and the leg considerably tumefied. It was through one of the fistulous openings that the blood flowed.

It was obviously a case of aneurism which had burst from coming in contact, most likely with the sequestrum. As the hemorrhage had been suppressed by the aid of compression I concluded to continue it. which was done by means of a roller applied from the toes upward with compresses over the openings. This dressing was carefully renewed and re-applied from day to day, the patient was very comfortable, and no bleeding took place till the 25th, in the night of which she lost several ounces of blood, which was arrested by additional compression. On the afternoon of the 26th, the hemorrhage again recurred and the patient was reduced very low.

It was then determined to tie the femoral artery, the patient not being in a state to support compression upon it sufficient to interrupt the circulation for want of sufficient intelligence. Indeed it is doubtful whether the case was a suitable one to be treated by that method.

By the aid of Professor Evans and several medical students, I placed a ligature around the artery on the evening of the 26th, which was done at the middle of the thigh without difficulty.

At 6 o'clock, P. M. one hour after the operation she complained of "numbness and fainting," foot and leg colder than the other, knee and thigh warmer, as had been the case for sometime before the operation.

27th.—Patient very comfortable, heat of leg restored, foot still cold. 29th—Limb a little warmer than the other with slight febrile reaction.

She continued to do well until March 5th, when a slight hemorrhage took place from one of the fistulous openings for which a roller and compress were applied.

This did not recur, and the patient set up, sewed, and even moved about notwithstanding directions to the contrary. March 16th.—Ligature came away and the wound was nearly healed. March 25th.—She was sufficiently recovered to remove from the hospital and is pursuing, at the present time, her usual mode of life.

